# Lack of IL-17 Receptor A signaling aggravates lymphoproliferation in C57BL/6 lpr mice

**DOI:** 10.1038/s41598-019-39483-w

**Published:** 2019-03-11

**Authors:** Odilia B. J. Corneth, Fleur Schaper, Franka Luk, Patrick S. Asmawidjaja, Adriana M. C. Mus, Gerda Horst, Peter Heeringa, Rudi W. Hendriks, Johanna Westra, Erik Lubberts

**Affiliations:** 1 Department of Rheumatology, Erasmus MC, University Medical Center Rotterdam, Rotterdam, The Netherlands; 2 Department of Pulmonary Medicine, Erasmus MC, University Medical Center Rotterdam, Rotterdam, The Netherlands; 3 Department of Rheumatology and Clinical Immunology, University Medical Center Groningen, University of Groningen, Groningen, The Netherlands; 4 Department of Pathology and Medical Biology, University Medical Center Groningen, University of Groningen, Groningen, The Netherlands; 5 Present Address: Department of Internal Medicine, division of Nephrology and Transplantation, Erasmus MC, University Medical Center Rotterdam, Rotterdam, The Netherlands

## Abstract

Defects in Fas function correlate with susceptibility to systemic autoimmune diseases like autoimmune lymphoproliferative syndrome (ALPS) and systemic lupus erythematosus (SLE). C57BL/6 lpr (B6/lpr) mice are used as an animal model of ALPS and develop a mild SLE phenotype. Involvement of interleukin-17A (IL-17A) has been suggested in both phenotypes. Since IL-17 receptor A is part of the signaling pathway of many IL-17 family members we investigated the role of IL-17 receptor signaling in disease development in mice with a B6/lpr background. B6/lpr mice were crossed with IL-17 receptor A deficient (IL-17RA KO) mice and followed over time for disease development. IL-17RA KO/lpr mice presented with significantly enhanced lymphoproliferation compared with B6/lpr mice, which was characterized by dramatic lymphadenomegaly/splenomegaly and increased lymphocyte numbers, expansion of double-negative (DN) T-cells and enhanced plasma cell formation. However, the SLE phenotype was not enhanced, as anti-nuclear antibody (ANA) titers and induction of glomerulonephritis were not different. In contrast, levels of High Mobility Group Box 1 (HMGB1) and anti-HMGB1 autoantibodies were significantly increased in IL-17RA KO/lpr mice compared to B6/lpr mice. These data show that lack of IL-17RA signaling aggravates the lymphoproliferative phenotype in B6/lpr mice but does not affect the SLE phenotype.

## Introduction

Defects in Fas function correlate with susceptibility to several systemic autoimmune diseases such as autoimmune lymphoproliferative syndrome (ALPS) and systemic lupus erythematosus (SLE)^1^.

ALPS is characterized by immune dysregulation due to an inability to regulate lymphocyte homeostasis through abnormalities in lymphocyte apoptosis or programmed cell death. This defect leads to lymphoproliferative disease with clinical manifestations that can include lymphadenopathy, splenomegaly, increased risk of lymphoma and autoimmune disease. Another hallmark for ALPS is expansion of T cells that express the alpha/beta T cell receptor but lack CD4 and CD8 (double negative (DN) T cells) in peripheral blood and tissue^2^. Moreover, lupus prone mice, often present with an ALPS like phenotype.

SLE is a systemic autoimmune disease that is characterized by accumulation of large numbers of DN T cells and production of multiple autoantibodies, mostly directed against nuclear antigens. Lupus nephritis is one of the most serious manifestations^3^. The cause of the disease is unknown, but both genetic and environmental factors play a role. Anti-nuclear antibodies (ANAs) and immune complexes can promote T cell activation and the production of interferon (IFN) type I by plasmacytoid dendritic cells (pDCs)^4,5^. IFN type I expression (signature) is increased in approximately half of SLE patients^6^. Monocyte derived DCs (mDCs) are activated by IFN type I and can induce T helper cell differentiation, enhanced B cell activation and survival through B cell activating factor (BAFF) production^7^. This leads to the production of auto-reactive antibodies, mostly ANAs, which form immune complexes and deposits in small capillaries in for example the kidneys, inducing inflammation and tissue damage^8^. Tissue damage, or disturbed clearance of apoptotic cells, can lead to the release of damage associated molecular pattern (DAMP) molecules, such as High Mobility Group Box-1 (HMGB1). HMGB1 can be released from activated, apoptotic and necrotic cells. Recently it was demonstrated that HMGB1 levels are increased in serum and urine of SLE patients and are related to disease activity^9,10^.

Both ALPS and SLE phenotypes can spontaneously develop in lpr mouse models, carrying mutations in the *Tnfrsf6* gene, encoding the Tumor Necrosis factor (TNF)-family receptor protein FAS or CD95 that has the capacity to induce apoptosis. These lpr mice are often referred to as lupus mice and display lymphoproliferation, expansion of DN T cells and autoimmune nephritis, including anti-dsDNA autoantibodies^11^. It is known that MRL/lpr mice are severely affected and develop autoimmune manifestations that both serologically and pathologically show similarities to human SLE but also demonstrate the lymphoproliferative phenotype that characterizes ALPS^12^. C57BL/6-lpr (B6/lpr) mice are also used as an animal model of ALPS and present a milder SLE phenotype^13^.

Interleukin 17A (IL-17A) is a pro-inflammatory cytokine implicated in different autoimmune disorders^14–16^ and can be produced by several immune cells, including several T cell subsets such as T helper 17 (Th17) cells, CD8+ T cells, natural killer (NK) cells, and DN T cells^17,18^. IL-17A is a member of the IL-17 cytokine family and signals through a heterodimeric receptor complex composed of the IL-17 receptor A (IL-17RA) and IL-17RC subunits^19^. The IL-17RA subunit appears to be the common receptor subunit for most if not all IL-17 cytokine family members although the signalling pathways for IL17B and IL-17D are not fully elucidated^19^. Maintenance of Th17 cells, the predominant producers of IL-17 in autoimmunity, depends on IL-23 signalling^20^.

Several lines of evidence indicate that IL-17A is involved in ALPS and SLE pathology. In addition, IL-17F and IL-17C promoted Th17 cell-driven glomerular inflammation and tissue injury^21,22^. IL-17A inhibits Fas-induced cell death and its neutralization enhances lymphocyte apoptosis in patients with ALPS. In MRL/lpr mice anti-IL-17A antibody treatment ameliorates the autoimmune manifestations and to a lesser extent the lymphoproliferative phenotype, prolonging survival of MRL/lpr mice^12^. In SLE patients, IL-17A plasma levels and numbers of IL-17A producing peripheral blood mononuclear cells (PBMCs) are reported to be increased and correlate with disease severity^23–25^. Furthermore, IL-17A producing DN T cells have been found in the kidneys of lupus nephritis patients^17^. However, there are also studies in SLE patients that do not observe a relation between IL17A and disease activity^26,27^.

In murine models of SLE increased levels of IL-17A, and IL-23 receptor-positive cells have been observed^28,29^. It was demonstrated that B6/lpr mice lacking IL-23 receptor (IL-23R) signalling were completely protected against SLE development^30^. These mice had decreased numbers of IL-17A producing cells in the lymph nodes and decreased anti-DNA antibody production, suggesting a crucial role for the IL-23/IL-17A axis in SLE pathogenesis in this model^30^.

Since the IL-17RA subunit is a common receptor unit for most if not all IL-17 family members^19^, we investigated the phenotype in aging B6/lpr mice lacking IL-17RA. Our data show that B6/lpr mice lacking IL-17RA develop a severely lymphoproliferative phenotype without effecting ANA levels. Furthermore, an increase in serum HMGB1 was noted, indicating that activated immune cells might secrete HMGB1.

## Results

### Increased cell numbers in lymphoid organs of IL-17RA KO/lpr mice compared to B6/lpr mice

**Figure 1:**
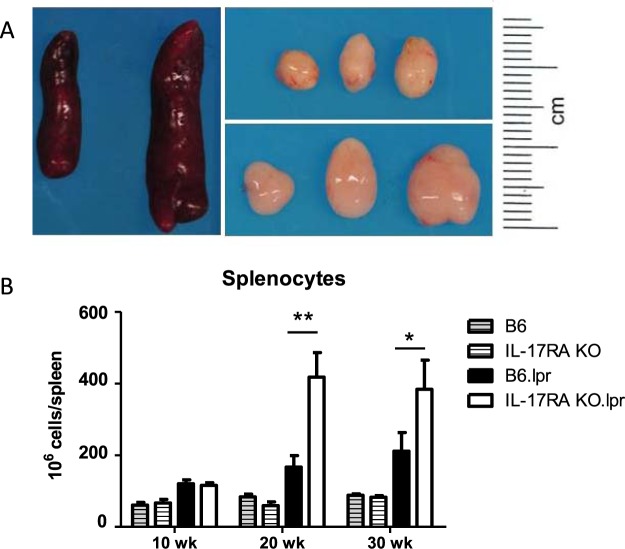
Increased spleen and lymph node size in IL-17RA KO/lpr mice. (**A**) Spleens and cervical lymph nodes from 30 week old B6/lpr (left and top) and IL-17RA KO/lpr (right and bottom). (**B**) Total number of splenocytes (all live cells in spleen) at different ages. Bars depict wild type B6 (grey stripe), wild type IL-17RA KO (open stripe), B6/lpr (black), and IL-17RA.KO/lpr (open) mice at 10, 20 and 30 weeks of age. Mean and SEM are shown for n = 3–21 mice per group; *p < 0.05; **p < 0.01.

B6/lpr and IL-17RA KO/lpr mice were followed over time and sacrificed at 10 weeks (no disease development in B6/lpr), 20 weeks (sub-clinical development of disease in B6/lpr mice) and 30 weeks of age (established disease in B6/lpr mice). At 30 weeks of age, the spleens and lymph nodes from IL-17RA KO/lpr mice were greatly enlarged compared to spleens and lymph nodes of B6/lpr mice (Fig. 1A). The total number of splenocytes was comparable between C57BL/6 (B6) wild-type and IL-17RA KO mice without the lpr mutation at 10, 20 and 30 weeks of age, and increased mildly over time in B6/lpr mice. However, the number of splenocytes in IL-17RA KO/lpr mice increased significantly over time to about twofold compared with B6/lpr mice at 30 weeks of age (Fig. 1B).

### Increased numbers of DN T and CD4+ T cells in IL-17RA KO/lpr mice

**Figure 2:**
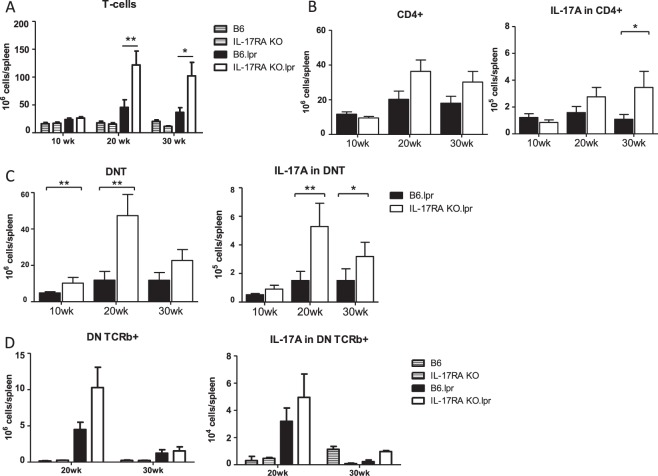
Expansion of T cell populations in IL-17RA KO/lpr mice. (**A**) Total numbers of splenic T cells (CD3+) in wildtype and lpr mice. Bars depict wild type B6 (grey stripe), wild type IL-17RA KO (open stripe), B6/lpr (black), and IL-17RA.KO/lpr (open) mice at 10, 20 and 30 weeks of age. (**B**) Total numbers of splenic CD4+ T cells (CD3+ CD4+ CD8−) (left) and IL-17A producing CD4+ cells(right) in B6/lpr (black bars) and IL-17RA KO/lpr (open bars). (**C**) Double negative (DN) splenic T cells (CD3+ CD4− CD8−) (left), and IL-17A producing DN T cells (right) in B6/lpr (black bars) and IL-17RA KO/lpr (open bars) mice at different ages. (**D**) Double negative (DN) splenic TCRβ+ (DN TCRb+) T cells (CD3+ CD4− CD8− TCRβ+) (left), and IL-17A producing DN TCRβ+ (IL-17A in DN TCRb+) T cells (right) for wild type and lpr mice at week 20 and 30. Mean is shown for n = 9–21 animals per group; *p < 0.05; **p < 0.01.

Both DN T and CD4+ T cells have been shown to play a role in the pathogenesis of SLE in patients^31^. To determine whether these subpopulations were increased in IL-17RA KO/lpr mice, we analyzed the splenic T cell compartment of these mice by flow cytometry. At weeks 20 and 30, an increase in the number of T cells was found in IL-17RA KO/lpr mice compared to B6/lpr mice (Fig. 2A). This was due to an increase in the total numbers of DN T cells, and to a lesser extent of CD4+ T cells (Fig. 2B,C). To further characterize the DN T cells, DN TCRβ+ analysis was performed. At week 20, higher numbers of DN TCRβ+ cells in B6.lpr were noted compared to C57BL/6 and IL-17RA KO mice which decline at week 30. In addition, the numbers of DN TCRβ+ are higher in the IL-17RA KO/lpr compared to B6.lpr mice at week 20 but not at week 30 (Fig. 2D). There was also an increase in numbers of IL-17A-producing DN T cells and CD4+ T cells in IL-17RA KO/lpr mice (Fig. 2B,C). At week 20, IL-17A+ DN TCRβ+ cells were elevated in b6.lpr and IL-17RA KO/lpr mice that decline to background levels at week 30 (Fig. 2D). These data indicate that lack of IL-17RA influenced the proliferation of CD4+ and especially DN T cells. The numbers of IFN-γ and IL-4 producing DN T cells and CD4+ T cells were similar in IL-17RA KO/lpr and B6/lpr mice (data not shown).

### Serum cytokine levels are increased in IL-17RA KO/lpr mice

**Figure 3:**
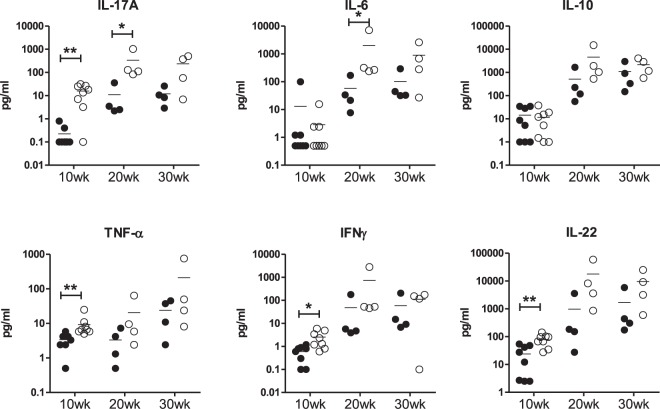
IL-17A, IL-6, IL-10, TNF-α, IFN-γ, and IL-22 levels in B6/lpr and IL-17RA KO/lpr mice. Serum cytokine levels as measured by multiplex at 10, 20 and 30 weeks of age in B6.lpr (black circles) and IL17RA KO/lpr (open circles); *p < 0.05; **p < 0.01.

To further asses the cytokine profile in B6.lpr and IL-17RA KO/lpr mice, serum cytokine levels were measured by multiplex assay. As expected, levels of IL-17A were significantly increased in IL-17RA KO/lpr mice at 10 weeks of age (Fig. 3). In addition, in young IL-17RA KO/lpr mice, IFN-γ, TNF-α and IL-22 were increased (Fig. 3). IL-6 was significantly increased at week 20, but surprisingly none of the other cytokines analysed were significantly different between the two groups of mice at week 30, despite the difference in disease phenotype at that age. No difference was observed for IL-4 (data not shown), and IL-10 (Fig. 3).

### Plasma cell formation is enhanced in IL-17RA KO/lpr mice

**Figure 4:**
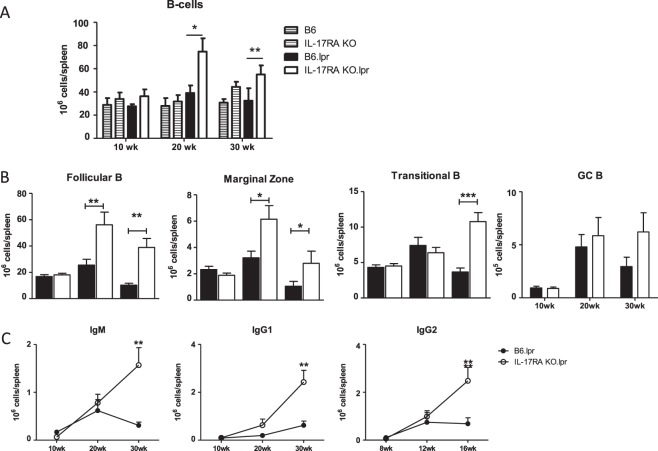
Expansion of B cell populations in IL-17RA KO/lpr mice. (**A**) Total numbers of splenic B cells (CB19+) in wildtype and lpr mice. Bars depict wild type B6 (grey stripe), IL-17RA KO (open stripe), B6/lpr (black), and IL-17RA.KO/lpr (open) mice at 10, 20, and 30 weeks of age. [B] Total numbers of splenic follicular B cells (CD19+ B220+ CD21− CD23+),marginal zone B cells (CD19+ B220+ CD21+ CD23−), transitional B cells (CD19+ B220+ CD21− CD23−), and germinal centre B cells (CD19+ B220+ PNA+ CD95+) in B6/lpr and IL-17RA KO/lpr mice at different ages. (**C**) Total numbers of IgM, IgG1 and IgG2 producing CD138+ plasma cells at different ages. Mean and SEM are shown for n = 9–21 animals per group; *p < 0.05; **p < 0.01; ***p < 0.001.

As we found increased IL-6 levels in serum of IL-17RA KO/lpr mice at early disease onset and increased B cell numbers in these mice (Fig. 4A), we further analyzed activation of the splenic B cell compartment. Analysis of CD21 and CD23 expression by flow cytometry^32^ showed that the increase in number of B cells could be attributed primarily to an increase in the number of follicular B cells (CD19+ B220+ CD21− CD23+) and marginal zone B cells (CD19+ B220+ CD21+ CD23−) (Fig. 4B), although at week 30 also the size of the transitional B cell fraction (CD19+ B220+ CD21− CD23−) was increased in IL-17RA KO/lpr mice (Fig. 4B). Analysis of germinal centre formation showed that after an initial rise at 20 weeks of age the numbers of germinal centre B cells (PNA+ CD95+ CD19+) were decreased in B6/lpr mice at 30 weeks of age, but not significantly lower than in IL-17RA KO/lpr mice (Fig. 4B). However, flow cytometry analysis for Ig subclasses showed that the numbers of IgM, IgG1 and IgG2 plasma cells present in the spleen at 30 weeks of age was significantly higher in IL-17RA KO/lpr mice compared with B6/lpr mice (Fig. 4C). Together, these data show increased B cell activation in the absence of IL-17RA signalling in B6/lpr mice.

### Autoimmune pathology is not enhanced in B6/lpr mice in absence of IL-17RA signalling

**Figure 5:**
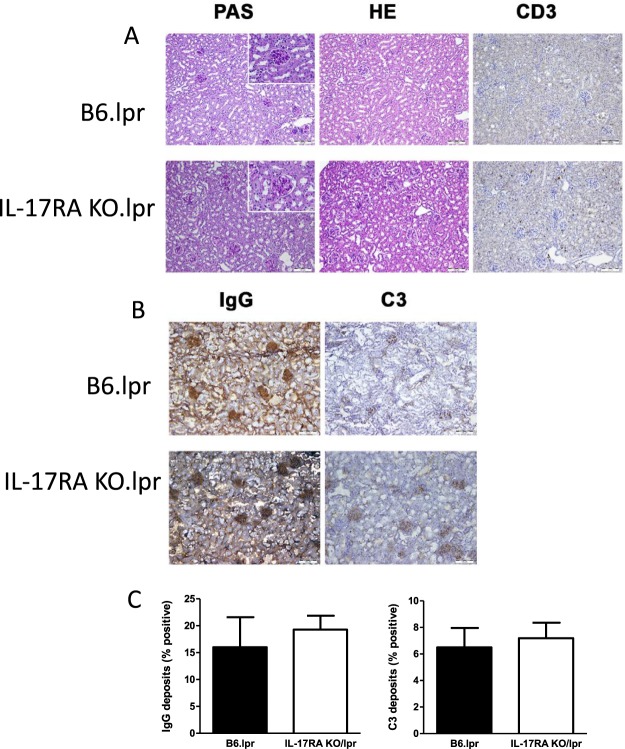
No induction of kidney damage in both IL-17RA KO/lpr and B6/lpr mice. (**A**) Representative PAS staining, HE staining, CD3 staining, in kidneys of 30 week old B6.lpr and IL-17R KO/lpr (10×). Inserts show a glomerulus in detail (40×). Representative pictures are shown for n = 6 animals per group. (**B**) Representative staining of C3 and IgG deposition in kidneys of 30 week old B6/lpr and IL-17R KO/lpr (10×). (**C**) Quantitative analysis of C3 and IgG deposition in kidney sections of 30 week old B6.lpr and IL-17R KO/lpr. Box and Whiskers plot, median and interquartile range are shown for n = 5–9 mice per group.

To investigate whether the increase in cytokine producing T cells and plasma cells in the spleen was associated with enhanced kidney pathology in IL-17RA KO/lpr mice, kidney slides were specifically stained and evaluated. By light microscopic analysis no thickening of the glomerular basal membrane in IL-17RA KO/lpr mice could be observed, indicating absence of kidney damage (Fig. 5A). Moreover, no increased influx of CD3-positive cells was seen in kidneys from IL17RA KO/lpr mice compared to B6/lpr mice (Fig. 5A). In addition, we found no enhanced C3 complement deposition and no enhanced IgG antibody deposition in kidneys sections from 26-week-old IL-17RA KO/lpr mice compared to B6/lpr (Fig. 5B,C). To further investigate renal inflammation, mRNA levels of the pro-inflammatory cytokine MCP-1 and the renal injury-related biomarkers NGAL and KIM-1 were assessed in kidneys. Moreover, mRNA levels of CD68 were measured to determine influx of macrophages. There was no significant difference in expression of NGAL and KIM-1 in kidneys of B6/lpr compared to IL17-RA KO/lpr compared, indicating no overt kidney damage in both groups (Table 1), confirming renal histology.

**Table 1:** IL-17RA KO does not affect renal mRNA levels of CD68, MCP-1, NGAL or KIM-1.

	B6/lpr	IL-17RA KO/lpr
CD68	0.0009 (0.0003–0.027)	0.007 (0–0.024)
MCP-1	0.0002 (0–0.003)	0.0005 (0–0.009)
NGAL	0.0001 (0–0.018)	0.001 (0–0.018)
KIM-1	0.0001 (0–0.0221)	0 (0–0.0221)

mRNA analysis in kidney tissue of b6/lpr and IL-17RA ko/lpr for CD68, MCP-1 (monocyte chemotactic protein-1), KIM-1 (kidney injury molecule-1), NGAL (neutrophil gelatinase-associated lipocalin). Data are presented as median (range) of relative expression compared to GAPDH (glyceraldehyde-3-phosphate dehydrogenase).

### HMGB1 levels in serum are increased in B6/lpr mice lacking IL-17RA signalling

**Figure 6:**
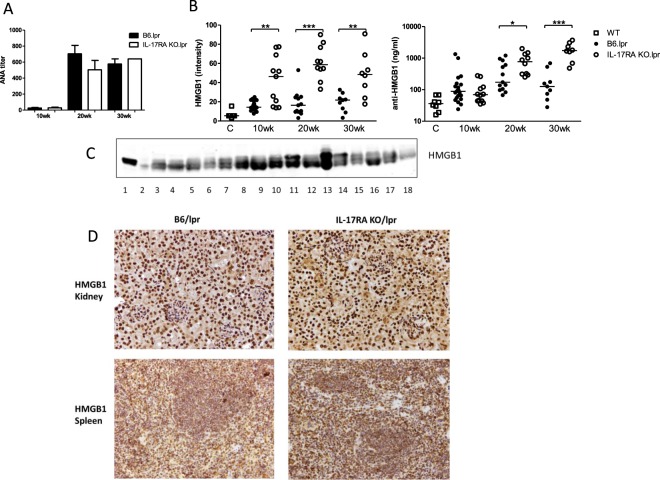
HMGB1 levels are increased in serum of IL-17RA KO/lpr mice. (**A**) Serum ANA titers by immunofluorescence for B6/lpr mice (black bars) and IL-17RA KO/lpr (open bars). Mean and SEM are shown for n = 5–9 animals per group. (**B**) Serum HMGB1 levels measured by Western Blot and serum anti-HMGB1 levels measured by ELISA in wildtype B6 mice, B6/lpr, and IL-17RA KO/lpr mice at different ages. Line indicates median. (**C**) Representative Western blot of HMGB1 in sera of lpr mice, 1 = positive HMGB1 control, 2 = Biorad molecular weight marker, 3–18 = mouse lpr sera. (**D**) Kidney and spleen HMGB1 staining 30 week old B6/lpr and IL-17R KO/lpr (10×). Representative pictures are shown for n = 6 animals per group. *p < 0.05; **p < 0.01; ***p < 0.001.

SLE is characterized by circulating ANA and consumption of complement C3. Complement C3 levels did not decrease over time in B6/lpr or IL-17RA KO/lpr mice (data not shown). Serum ANA titers increased with age in both groups (Fig. 6A), but ANA titers were not significantly higher in IL-17RA KO/lpr mice than in B6/lpr mice at any age. HMGB1 levels were determined in serum by Western blot (see Fig. 6C for a representable example of the Western blot). HMGB1 was low in control C57BL/6 wild type or IL-17RA KO mice (Fig. 6B). Remarkably, at 10 weeks of age HMGB1 levels were already increased in IL-17RA KO/lpr mice compared to B6/lpr mice (Fig. 6B). Furthermore, anti-HMGB1 antibody levels, which were low in C57BL6 and IL-17RA KO mice (Fig. 6B) increased in IL-17RA KO/lpr mice with age and reached a significantly higher level than in B6/lpr mice (Fig. 6B). To investigate the source of HMGB1, both kidney and spleen sections of (the same) mice were stained for HMGB1 (Fig. 6D). Similar nuclear HMGB1 expression, no cytoplasmic HMGB1 and no apparent release of extracellular HMGB1 due to activation were observed in kidneys of both IL-17RA KO/lpr and B6/lpr mice (Fig. 6D). In spleen sections however, we did observe extracellular HMGB1. Moreover, HMGB1 negative nuclei (blue staining) were present, which suggests active release of HMGB1 from these cells, but there was no difference in HMGB1 expression between IL-17RA KO/lpr and B6/lpr mice (Fig. 6D).

## Discussion

This study demonstrates that deletion of IL-17RA signaling markedly aggravates the lymphoproliferation in B6/lpr mice, but does not affect SLE pathology. The greatly increased spleen and lymph node size in IL-17RA KO/lpr mice, characterized by expansion of CD4+ T cells, CD8+ T cells, DN T cells and B cells indicates an important role for IL-17RA signaling in limiting the lymphoproliferative phenotype in B6/lpr mice. Remarkably however, neither serum ANA titers, antibody deposition in the kidney nor kidney damage were different between IL-17RA KO/lpr and B6/lpr mice, showing that these hallmarks of SLE are independent of IL-17RA signaling in this model. Furthermore, increased serum HMGB1 levels followed by elevated anti-HMGB1 antibody levels were seen in IL-17RA KO/lpr mice compared to B6/lpr mice.

A strong lymphoproliferative phenotype was found in IL-17RA KO/lpr mice leading to visible lymphadenomegaly. The phenotype was accompanied by increased lymphocyte numbers of which enhanced DN T cell proliferation was most prominent. Total numbers of IL-17A-cytokine producing DN T cells and CD4+ T cells were increased at 20 and 30 weeks of age in IL-17RA KO/lpr mice compared with B6/lpr mice. This increase is most likely a consequence of loss of a negative feedback loop limiting IL-17A production through IL-17RA signalling. This indicates that IL-17RA signaling plays a more significant role in generating negative feedback to regulate the IL-17 inflammatory response rather than functioning in effector signaling to aggravate or initiate disease in these mice. It might be that IL-17RA signaling can limit cell proliferation or survival of CD4+ and DN T cells in particular. In normal transgenic IL-17RA KO mice, loss of this regulatory function can apparently be rescued by other factors, however, additional loss of FAS-FASL signaling induces expansion of immune cells.

A role for IL-17RA signaling has been shown in disease progression in lupus prone BXD2 mice^33,34^. When crossed with IL-17RA KO mice, BXD2 mice are partially protected against disease development. This can be attributed to impaired germinal center reactions in these mice. In the present study, no decrease in the number of PNA+ germinal center B cells in the spleens of IL-17RA KO/lpr mice was found at any age. Instead, the number of germinal center B cells appeared to be slightly increased, albeit not significantly. Importantly, ANA titers are not decreased in IL-17RA KO/lpr mice, suggesting that in B6/lpr mice ANA development is IL-17RA independent. As BXD2 mice have a mixed C57BL/6 and DBA genetic background, it is possible that strain specific genes can explain these discrepancies, as normal germinal center formation in IL-17RA KO mice on a C57BL/6 background in collagen induced arthritis and after influenza infection were found^35^.

A recent study demonstrated that IL-17 signaling drives type I interferon induced proliferative crescentic glomerulonephritis (cGN) in lupus prone mice^36^. Impaired infiltration of alternatively activated macrophages into the kidney was observed. However, no signs of enhanced lymphoproliferative phenotype was reported in the IL-17RA deficient B6.lpr mice which may be due to the poly I:C induced TLR3 stimulation with enhanced type I interferon induction in this cGN lupus model before significant lymphoproliferation starts to develop spontaneously as we have shown in this study.

Schmidt *et al*. reported that IL-17A deficiency did not affect the morphologic or functional parameters in MRL/lpr mice with lupus nephritis, nor did IL-17A neutralization affect the clinical course of nephritis in NZB/NZW mice^37^, which is in line with our results.

In MRL/lpr mice, HMGB1 levels correlate with disease progression^38^. In the present study HMGB1 and anti-HMBG1 levels were significantly increased in lpr mice lacking IL-17RA while no difference in ANA levels were observed between IL-17R KO/lpr and B6/lpr mice. A morphological hallmark of human and experimental lupus nephritis is the trafficking of inflammatory cells into the kidneys which was not observed in both B6/lpr and IL-17RA KO/lpr mice during the time span of our study. Thus, in both B6/lpr and IL-17RA KOL/lpr mice there was no induction of lupus nephritis which suggests that there is no direct involvement of HMGB1 and potential immune complex formation with anti-HMGB1 in driving nephritis in these models. HMGB1 can be released from the spleen as was observed in IL-17RA KO/lpr mice. Subsequently, HMGB1 can have an effect on apoptosis of cells or interfere in phagocytosis of apoptotic cells^39–41^ which may partly explain the enhanced lymphoproliferative phenotype in IL-17RA KO/lpr mice. Importantly, in a sepsis induced mouse model it was shown that administration of recombinant HMGB1 induced splenomegaly, lymphocytosis and splenocyte priming^42^. Previously, a role for IL-23 and IL-23R signaling in the SLE phenotype of B6/lpr and MRL/lpr mice was reported^29,30,43^. In IL-23R KO/lpr mice, which do not develop lupus, the total number of DN T cells as well as the total number of IL-17A producing DN T cells and CD4+ T cells was decreased. This was accompanied by lower serum IgG and ANA levels, decreased IgG and complement deposition in the kidneys and absence of kidney damage^30^. These data suggest an important role for IL-17A in the IL-23 targeting approach of SLE although direct effects of IL-23 in disease development cannot be excluded.

By deletion of IL-17RA potentially several IL-17 family members are not functional as this subunit is involved in signaling of many if not all IL-17 family members. Therefore, this study goes beyond the role of IL-17A alone. Interestingly, IL-23 signaling is not deficient in the IL-17RA KO/lpr mice. It has been shown that CISK (Act1) knockout mice on a FcγRIIB background showed greatly improved survival and were largely protected from the development of glomerulonephritis^44^. CISK (Act1) is part of the IL-17 receptor signaling pathway and loss of CISK blocks signaling by all IL-17 cytokines and to a lesser extent loss of IL-17A^44^. However, BAFF can activate B cells and myeloid cells via the BAFF receptor to activate CISK (Act1), indicating that the CISK/Act1 KO may have a broader impact in different signaling pathways in the development of SLE than the IL-17RA KO/lpr in our study^14^. A proposed mechanism is that IL-17RA deficiency induces enhanced lymphoproliferation and that this leads to release of HMGB1 from activated or dying cells in spleens and lymph nodes, inducing a pro-inflammatory loop. However, evidence for this mechanism is not investigated in the present study. It is also possible that other IL-17 family member(s) are involved in controlling the lymphoproliferative phenotype in B6/lpr mice. Further studies are needed to identify these mechanisms. However, our data clearly demonstrate that IL-17RA signaling is involved in the regulation of the lymphoproliferative phenotype in B6/lpr mice indicating that caution should be taken to modulate IL-17RA signaling in lymphoproliferative prone individuals.

## Materials and Methods

### Mice

C57BL/6-lpr/lpr (B6/lpr) mice were purchased from The Jackson Laboratory, USA, and IL-17 receptor A knock-out (IL-17RA KO) mice on a C57BL/6 background were kindly provided by dr. J. Tocker, Amgen, Seattle, USA. For genotyping of the IL-17RA construct, 5′- CTTGTGTAGCGCCAAGTG, 5′-AGCTGCTGTTAGCACTTTGC and 5′- CGTACGCACACACTCTCGA primers were used. For genotyping of the lpr construct, 5′- GTAAATAATTGTGCTTCGTCAG, 5′-TAGAAAGGTGCACGGGTGTG and 5′- CAAATCTAGGCATTAACAGTG primers were used. The mouse lines were crossed to generate IL-17RA KO B6/lpr mice. Mice were housed under SPF conditions in the Erasmus Medical Center Animal Facility (EDC) and provided with food and water *ad libitum*. All experiments were approved by the Erasmus MC Animal Ethical Committee (DEC) and were performed according to strict governmental and international guidelines on animal experimentation. Mice were sacrificed at the age of 10, 20 or 30 weeks. Blood was drawn for collection of serum and spleens, kidneys and cervical lymph nodes were harvested.

### Flow cytometry for B- and T cells

Spleens were harvested and single cell suspensions prepared using 100 µm filters. Flow cytometry for B and T cells was performed as previously described^35^. Anti-CD19, anti-B220, anti-CD21, anti-CD23, anti-IgM, anti-CD3, anti-CD4, anti-CD8, anti-IL-17A, anti-IL-4 and anti-IFN-γ antibodies were obtained from eBioscience (San Diego, CA, USA), anti-IgD, anti- CD95, anti-CD138, anti-IgG1 and anti-IgG2ab antibodies from BD BioSciences, anti-IL-10 antibody was purchased from Biolegend (San Diego, CA, USA) and biotinylated peanut agglutinin (PNA) from Sigma-Aldrich (St Louis, USA).

Samples were measured on a FACS Canto II HTS or a LSR II flow cytometer (BD BioSciences) and analysis was performed using FlowJo v7.6 research software (Tree Star Inc. Ashland, OR).

### Histology

Kidney tissue samples from 30 week-old female IL-17RA KO lpr and B6/lpr mice were frozen in Tissue-Tec O.C.T. Compound (Sakura Finetek Europe B.V) and stored at −80 °C or embedded in formalin. Two µm sections of formalin-fixed, paraffin-embedded kidney tissues were cut and were routinely stained with haematoxylin or eosin (H&E) and periodic acid- Schiff (PAS) for evaluation of kidney pathology. Complement C3 and IgG staining was performed on 5 µm frozen kidney sections with 1 µg/ml rabbit anti-C3 antibody (Thermoscientific) followed by goat-anti-rabbit IgG-HRP (Dako). For IgG staining rabbit anti-mouse IgG-HRP (Dako) was used. Peroxidase activity was detected with DAB and sections were counterstained with Mayer’s hematoxylin. All sections were scored digitally after examination using a Nanozoomer Digital Pathology Scanner (NDP Scan U10074-01, Hamamatsu Photonics K.K., Japan) and quantified ((number of positive pixels* 0.5) + number of strong positive pixels/total pixels) with software of ImageScope Viewer (V11.2.0.780 Aperio, e-Pathology Solution, CA, USA).

HMGB1 and CD3 staining was performed on 2 µm paraffin sections using polyclonal anti-HMGB1 (Abcam, Cambridge, UK) and polyclonal anti-CD3 (Dakocytomation).

### RNA isolation

Total RNA was extracted from 20 10 µm thin kidney cryo-sections using RNeasy Mini plus Kit (Qiagen, Westburg, Leusden, The Netherlands) according to the manufacturer’s instructions. Integrity of RNA was determined by agarose gel electrophoresis. RNA quantity (OD-260) and quality (OD-260/OD-280) were determined using an ND-1,000 UV-Vis spectrophotometer (NanoDrop Technologies, Rockland, DE). cDNA was synthesized from 1 µg RNA using M-MLV Reverse Transcriptase and oligo(dT) 24 (Life Technologies, USA). mRNA expression of IFN-γ, TNF-α, IL-6, HMGB1, MCP-1 (monocyte chemotactic protein-1), KIM-1 (kidney injury molecule-1), NGAL (neutrophil gelatinase-associated lipocalin) and GAPDH (glyceraldehyde-3-phosphate dehydrogenase) was measured by the real-time quantitative PCR system (ABI Prism 7900HT Sequence Detection System, Applied Biosystems, USA) with specific Taqman probes. The amount of target was normalized to an endogenous reference (GAPDH) and expressed as relative expression (2^−ΔCT^).

### Serum analysis

ANA titers were measured by immunofluorescence on HEp-2000 coated glass slides (Biomedical Diagnostics, Eindhoven, the Netherlands), using serial dilutions of mouse serum in PBS, and rabbit anti-mouse IgG-FITC (Dako, Glostrup, Denmark) for detection. Complement levels were measured using a commercial ELISA, according to the manufactures instructions (GenWay Biotech, San Diego, USA).

Serum cytokine levels were quantified with a Multiplex panel (ProcartaPlex Mouse Simplex; Affymetrix eBioscience, Vienna, Austria) according to the manufacturer’s instructions. Samples were measured on a Luminex 100 System (Luminex, Austin, TX, USA) and data was analyzed with StarStation software, version 2.3 (AppliedCytometry, Birmingham, UK). The following cytokines were assessed: IL-4, IL-6, IL-10, IL-17A, IL-22, IFN-γ and TNF-α.

HMGB1 levels were measured by Western Blotting as described previously for human serum^9^. Detection of HMGB1 on blots was performed with polyclonal anti-HMGB1-biotin (Thermoscientific, Etten-Leur, the Netherlands), and streptavidin-IRDye800 (LI-COR Biotechnology, Lincoln, NE, USA). Blots were scanned with an Odyssey infrared Imaging System (LI-COR Biotechnology) and analyzed with Odyssey software. In each blot a cell lysate made of Jurkat cells was run as a standard. HMGB1 levels were presented as the fluorescence intensity against the standard.

Levels of anti-HMGB1 were measured by in-house ELISA. Costar polystyrene plates were coated with 1 µg/ml recombinant HMGB1 (Sigma, St. Louis, MO, USA) and mouse sera were added in dilutions of 20 and 80 times. Detection of antibodies was done with rabbit anti- mouse IgG-HRP (Dako, Glostrup, Denmark) and TMB color reaction. Levels of anti-HMGB1 were calculated against a standard curve of a monoclonal anti-HMGB1 (R&D systems, Abingdon, United Kingdom).

### Statistical analysis

Data was analyzed using Prism software v5.04 (GraphPad Software Inc. La Jolla, CA). For comparisons, a non-parametric Mann-Whitney U test was used. P-values < 0.05 were considered significant.

## Supplementary information

Representative Western blot of HMGB1 in sera of lpr mice.
