# Supplementary material for: Lack of IL-17 Receptor A signaling aggravates lymphoproliferation in C57BL/6 lpr mice
Source: Sci Rep. 2019 Mar 11;9:4032. doi: 10.1038/s41598-019-39483-w (PMC6412096; doi:10.1038/s41598-019-39483-w)
Supplement: Supplementary file 1 — Representative Western blot of HMGB1 in sera of lpr mice. [file 41598_2019_39483_MOESM1_ESM.pdf]

# Lack of IL-17 Receptor A signaling aggravates lymphoproliferation in C57BL/6 lpr mice

Odilia B.J. Corneth[1,2, ‡]; Fleur Schaper[‡,3]; Franka Luk[1,5]; Patrick S. Asmawidjaja[1]; Adriana M.C. Mus[1]; Gerda Horst[3]; Peter Heeringa[4]; Rudi W. Hendriks[2], Johanna Westra[3] and Erik Lubberts[1*]

[1]Department of Rheumatology, Erasmus MC, Rotterdam, The Netherlands

[2]Department of Pulmonary Medicine, Erasmus MC, Rotterdam, The Netherlands

[3]Departments of Rheumatology and Clinical Immunology[illegible]

[4]Pathology and Medical Biology, University Medical Center Groningen, Groningen, The Netherlands

5Current address: Nephrology and transplantation, Department of Internal Medicine, Erasmus MC, Rotterdam, The Netherlands

[‡]Authors contributed equally

**Short title:** IL-17RA signaling limits lymphoproliferation in C57BL/6-lpr mice

This project was funded by the Dutch Arthritis Foundation (Reumafonds, grant no DAA 0801043).

**Correspondence to:** Erik Lubberts, PhD, Department of Rheumatology, Erasmus University Medical Center, Wytemaweg 80, 3015 CN, Rotterdam, The Netherlands. Phone: +31-10-7044309. Email: E.Lubberts@erasmusmc.nl

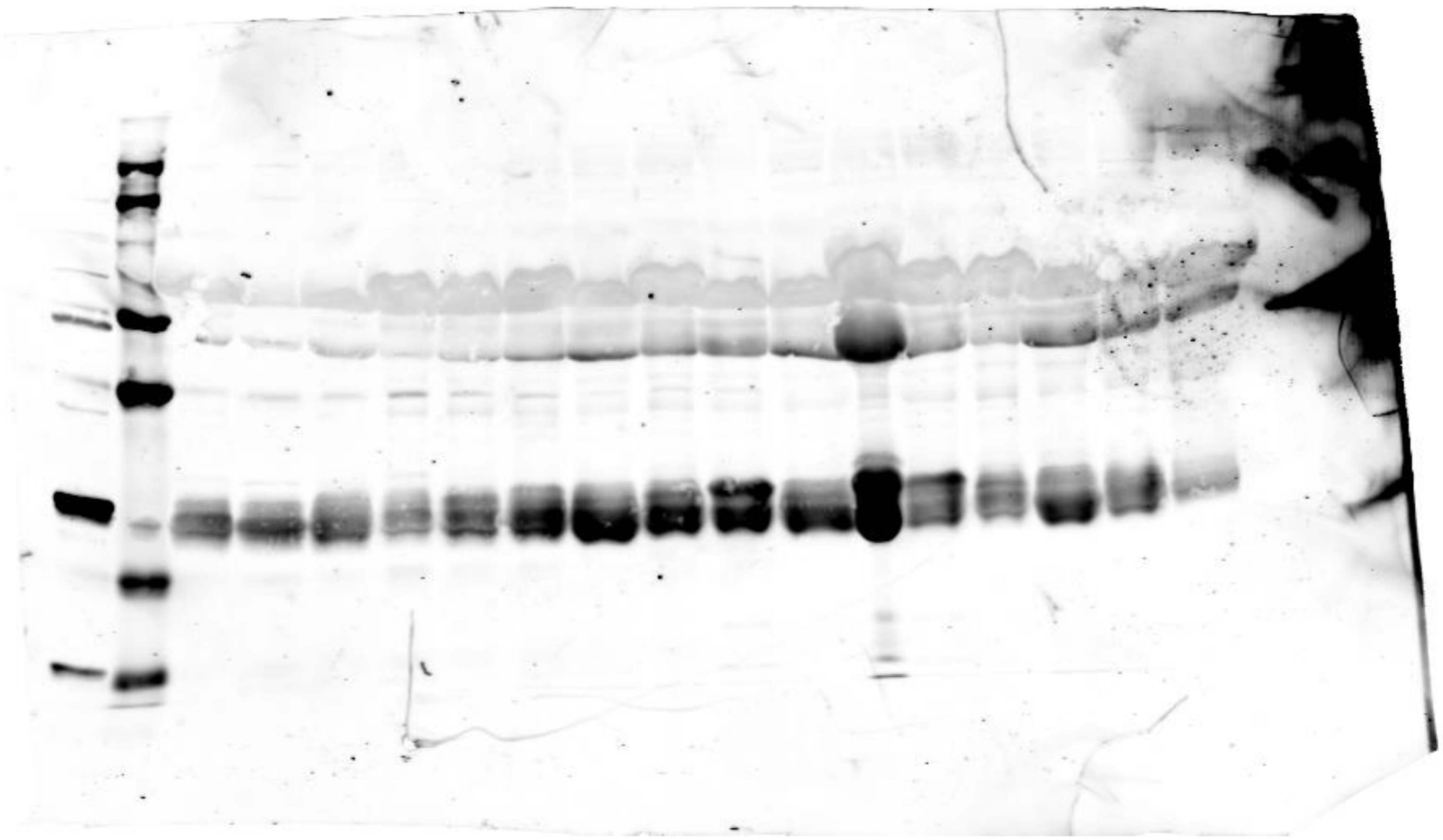

Suppl. Figure 1. Representative Western blot of HMGB1 in sera of lpr mice, 1=positive HMGB1 control, 2=Biorad molecular weight marker, 3-18 mouse lpr sera.
